# Early Intervention in Herpes Simplex Virus-1 Replication in Vitro with Allenic Macrolide Archangiumide

**DOI:** 10.3390/ijms26041537

**Published:** 2025-02-12

**Authors:** You Li, Jia-Qi Hu, Wen-Hai Feng, Changsheng Wu, Li Gao

**Affiliations:** 1 State Key Laboratory of Animal Biotech Breeding, Frontiers Science Center for Molecular Design Breeding, Ministry of Agriculture Key Laboratory of Soil Microbiology, Department of Microbiology and Immunology, College of Biological Sciences, China Agricultural University, Beijing 100193, China; 2 State Key Laboratory of Microbial Technology, Institute of Microbial Technology, Shandong University, Qingdao 266237, China; 3 National Key Laboratory of Veterinary Public Health and Safety, College of Veterinary Medicine, China Agricultural University, Beijing 100193, China; 4 Key Laboratory of Animal Epidemiology of the Ministry of Agriculture, College of Veterinary Medcine, China Agricultural University, Beijing 100193, China

**Keywords:** HSV-1, archangiumide, antiviral

## Abstract

Archangiumide is a unique macrolide natural product that features an endocyclic allene functionality, rendering it a prototype of a new class of secondary metabolites of microbial origin. However, its biological and/or pharmaceutical roles remain obscure. In this study, we have unveiled an antiviral potency of archangiumide that was effective against herpes simplex virus (HSV-1) replication. We found that archangiumide did not affect host cell viability, nor pathogen infectivity, but suppressed HSV-1 early replication, in terms of early replication genes, such as *ICP0*, *ICP4*, etc. Further scrutinizing the underlined master regulator, we found that HSV-1 VP16 protein expression was inhibited by archangiumide, as well as VP16 nuclear translocation. As VP16 is a coactivator of transcription, archangiumide harnessed the master regulator of HSV-1 early replication. Together, here we provide evidence that allene macrolide archangiumide possesses robust antiviral functions that may be valuable for a novel viral infection intervention, as macrolides are generally safe drugs for prolonged treatments.

## 1. Introduction

Since its first discovery in 1950s, macrolide has been one of the most commonly used families of antibiotics prescribed to treat infections of gram-positive bacteria. For example, azithromycin, clarithromycin, and erythromycin are commonly used to treat infections like pneumonia, sinusitis, pharyngitis, and tonsillitis [1]. Due to its excellent efficacy and safety record in clinical applications, as well as emerging multiple or extreme resistance, interest in exploring novel macrolides is vibrant for both natural microbial products and synthetic chemistry [2,3,4]. We identified archangiumide, a novel macrolide natural product produced by the myxobacterium Archangium violaceum SDU8, through a combination of genome mining and NMR-based metabolomic profiling. It is an unprecedented 19-membered allenic macrolide with a molecular formula of C_19_H_26_O_6_ [5]. Its structure is characterized by the presence of an endocyclic allene, two additional E-configured alkenes, and an annulated tetrahydrofuran ring. This complex architecture not only provides a significant synthetic challenge [6] but also endows the molecule with unique chemical properties. Under long term storage in particular conditions of illumination and room temperature, it undergoes gradual degradation. Preliminary biological activity assays demonstrated that archangiumide did not exhibit significant antitumor activity against over 20 common human tumor cells, neither antibacterial activity against Staphylococcus aureus, Bacillus subtilis, and Escherichia coli, nor antifungal activity against Candida albicans. In addition, it does not possess antioxidant (DPPH radical-scavenging) or anti-inflammatory (LPS-induced NO production in adherent cells) effects, yet its unique structure and the challenges associated with its synthesis make it a compelling candidate for further investigation in terms of therapies [5].

Although a plethora of reports has established the antibacterial functions of macrolides for decades, the long-neglected antiviral effects are now brought to spotlight, with the prominent example of azithromycin, which has been used to combat COVID-19 infection [7,8,9]. The exploration of macrolides as antiviral agents is further supported by the discovery of compounds like balticolid [10], a plecomacrolide with anti-HIV and anti-herpes simplex type I activity. These findings underscore the versatility of macrolide compounds in targeting different viral pathogens and suggest a broader application in antiviral strategies. Herpes simplex virus type 1 (HSV-1), a widespread human pathogen, is known to cause a spectrum of diseases ranging from mild oral and facial mucocutaneous infections to more severe and life-threatening conditions such as keratitis, encephalitis, and meningitis [11,12,13]. The virus’s ability to establish lifelong latent infections and its increasing drug resistance highlight the urgency for novel antiviral agents [14].

In this study, we embarked on the exploration of archangiumide for antiviral therapy, specifically targeting the mechanisms by which HSV-1 infects human cells. By meticulously dissecting the infection route of HSV-1, we aimed to pinpoint archangiumide’s intervention at early stages of viral infection. This approach aligns with the growing body of research that underscores the importance of early intervention in viral diseases, which is crucial for limiting viral replication and associated pathologies. As well, this research could pave the way for new treatment strategies against HSV-1 and contribute to the broader understanding of antiviral drug development.

## 2. Results

### 2.1. Archangiumide Significantly Suppressed HSV-1 Replication

Upon the initial discovery of archangiumide, we were not able to identify its bioactivity, as it has no antibacterial function as expected [5]. Inspired by azithromycin’s effectiveness in COVID-19, here we attempt to explore the antiviral activity of archangiumide. To sail in well-charted waters, we chose HSV-1 as our model pathogen, as its infection route has been mapped in detail and HSV-1 related tests are robust as well as bio-safety accessible.

**Figure 1:**
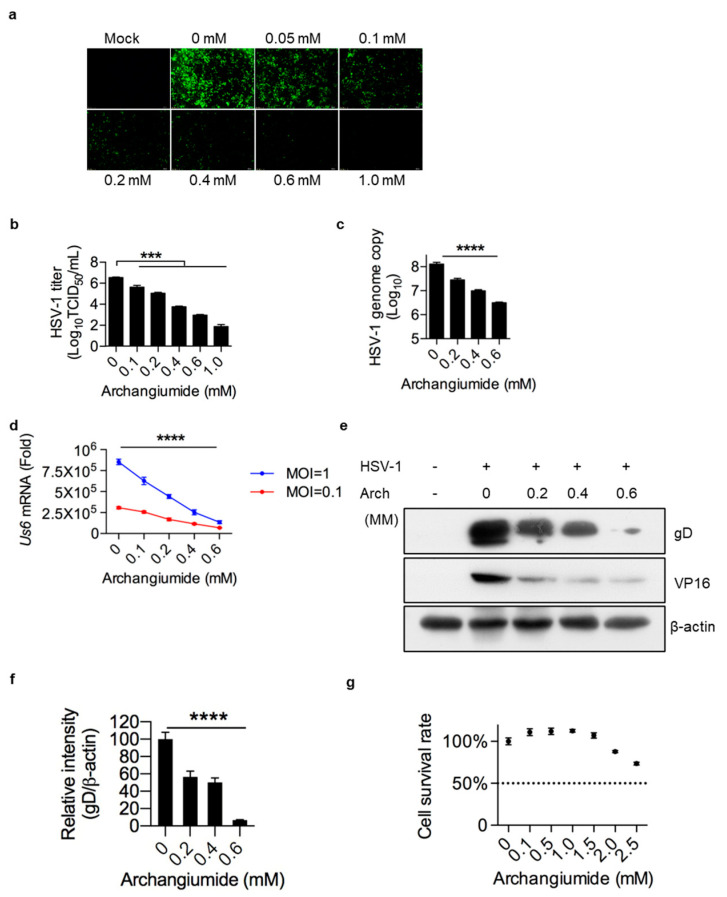
Archangiumide suppressed HSV-1 replication. (**a**,**b**) Archangiumide was added to HSV-1-eGFP virus-infected HeLa cells (MOI = 0.5) and cultured for 24 h. Sham infection was performed as control (Mock). GFP signal was captured (**a**). Fluorescent images were in 200× magnification. HSV-1 titer (TCID50/mL) was analyzed in culture supernatant after 24 h (**b**). Statistics were analyzed with non-parametric one-way ANOVA (Kruskl–Wallis test). ***, *p* < 0.001. n = 3. (**c**) Archangiumide was added to infected cells (MOI = 0.1). After 24 h culture, genomice DNA was extracted and the HSV-1 genome was analyzed by qPCR. Kruskl–Wallis one-way ANOVA. ****, *p* < 0.0001. n = 3. (**d**) gD transcription (Us6 mRNA) was analyzed by qPCR under high and low dose infections (MOI = 1 and MOI = 0.1). Kruskl–Wallis one-way ANOVA for each MOI group. ****, *p* < 0.0001. n = 3. (**e**) Western of gD and VP14 protein under archangiumide. β-actin as control. (**f**) Quantification of e.****, *p* < 0.0001. n = 3. (**g**) Cell survival rate under different doses of archangiumide. Dashed line represents CC50. n = 3. Data shown represent three independent experiments.

To monitor archangiumide’s effect on HSV-1 replication, GFP-tagged HSV-1 viruses were infected for 24 h at MOI 0.5, with or without archangiumide. As shown in Figure 1, archanguimide suppressed HSV-1 replication in a dose-dependent manner, in terms of GFP signal intensity (Figure 1a), viral load (Figure 1b), and viral genome copy (Figure 1c). Moreover, archanguimide suppressed HSV-1 glycoprotein D (gD, Us6 mRNA) transcription at higher and lower levels of viral infection (MOI = 1 and MOI = 0.1), confirming previous data (Figure 1d). In terms of protein level, gD and VP16 proteins were also suppressed (Figure 1e,f). Last but not least, archanguimide had low toxicity to cells (Figure 1g), and these findings were not due to host cell survival issues. Inferring from the virus titer (Figure 1b), archanguimide had an IC50 of 0.039 mM against HSV-1. A 5.75 mM CC50 (50% cytotoxicity concentration) is also inferred from Figure 1f. Therefore, archanguimide has a suitable therapeutic index of 147 (CC50/IC50) for HSV-1 intervention.

### 2.2. Archangiumide Was Not Able to Inactivate HSV-1 Directly

**Figure 2:**
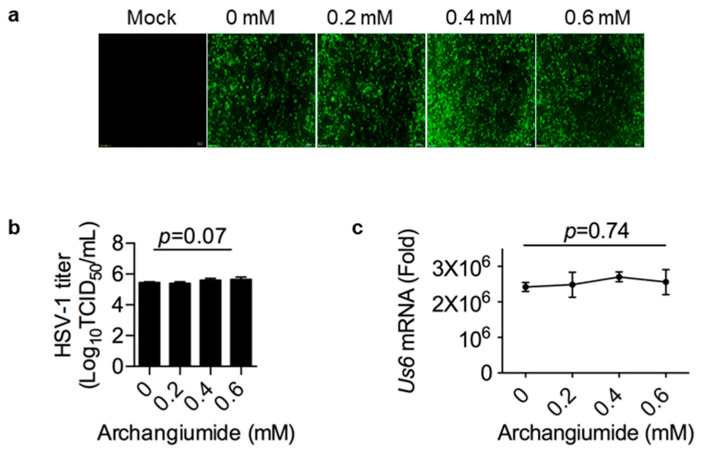
Archangiumide showed no effect on virus inactivation. Archangiumide was pre-incubated with HSV-1-eGFP virus stock for 2 h, then the stock was diluted into MOI = 0.1 to infect cells. (**a**) GFP signal was captured at 24 h. Fluorescent images were in 200× magnification. (**b**) HSV-1 titer (TCID50/mL) was analyzed in culture supernatant after 24 h. Kruskl–Wallis one-way ANOVA. n = 3. (**c**) gD transcription (Us6 mRNA) was analyzed by qPCR after 24 h. Kruskl–Wallis one-way ANOVA. n = 3. Data shown represent three independent experiments.

To understand the mechanism of archangiumide’s antiviral effect, we first tested whether archangiumide directly inactivated the pathogen. We pre-incubated HSV-1 stock with archangiumide for 2 h, then diluted the stock to infect cells. As shown in Figure 2a–c, archangiumide pre-incubation did not affect HSV-1 infection, not even at a dose of 0.6 mM. Thus, archangiumide was not able to inactivate HSV-1 directly, and its antiviral effect had impacts on the host cells rather than the pathogen.

### 2.3. Archangiumide Did Not Block HSV-1’s Attachment to the Cell, nor Penetration

**Figure 3:**
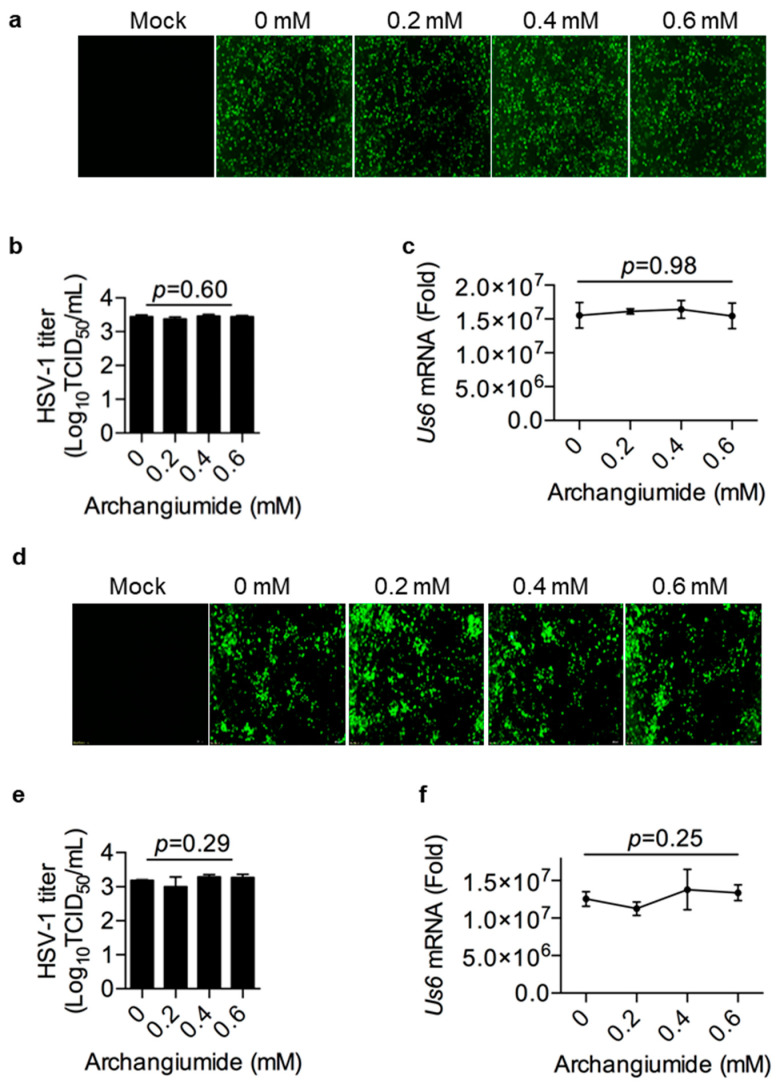
Archangiumide showed no effects on viral attachment and penetration. (**a**–**c**) With or without archangiumide, HSV-1 (MOI = 0.01) was loaded onto cells at 4 °C for 2 h for attachment, then removed. Infected cells were cultured in fresh medium and the GFP signal was captured at 24 h (**a**). Fluorescent images were in 200× magnification. (**b**) HSV-1 titer (TCID50/mL) was analyzed in culture supernatant after 24 h. Kruskl–Wallis one-way ANOVA. n = 3. (**c**) gD transcription (Us6 mRNA) was analyzed by qPCR after 24 h. Kruskl–Wallis one-way ANOVA. n = 3. (**d**–**f**) HSV-1 (MOI = 0.01) was loaded onto cells at 4 °C for 2 h for attachment, then removed. With or without archangiumide, attached HSV-1 was stimulated to penetrate at 37 °C for 15 min. After using an acidic PBS wash (pH = 3) to remove non-penetrated virus then neutralizing cells with a basal PBS wash (pH = 11), infected cells were cultured 24 h for analysis. (**d**) GFP signal was captured at 24 h. Fluorescent images were in 200× magnification. (**e**) HSV-1 titer (TCID50/mL) was analyzed in culture supernatant after 24 h. Kruskl–Wallis one-way ANOVA. n = 3. (**f**) gD transcription (Us6 mRNA) was analyzed by qPCR after 24 h. Kruskl–Wallis one-way ANOVA. n = 3. Data shown represent three independent experiments.

HSV-1 followed a classical virus–host interaction route, in terms of attachment to the host cell, penetration into the cell, release of the genome, replication of the genome, etc. [12,13]. Therefore, step by step, we scrutinized archangiumide’s intervention in HSV-1’s infection route. First, we tested archangiumide’s effect on viral attachment to the cell. HSV-1 was allowed to attach to pre-chilled cells for 4 h at 4 °C with or without archangiumide. Then, the infected cells were cultured in fresh medium for 24 h. As shown in Figure 3a–c, archangiumide did not suppress HSV-1 attachment to cells. Next, we tested archangiumide’s effect on viral penetration into cells. After HSV-1 attachment to the pre-chilled cell, the viral medium was removed, then the attached HSV-1 was allowed to penetrate cells at 37 °C for 15 min with or without archangiumide. Unpenetrated HSV-1 was removed with an acidic PBS wash, and infected cells were cultured in fresh medium for 24 h. As shown in Figure 3d–f, archangiumide did not suppress HSV-1 penetration into cells either.

### 2.4. Archangiumide Suppressed Early Replication of Intracellular HSV-1

**Figure 4:**
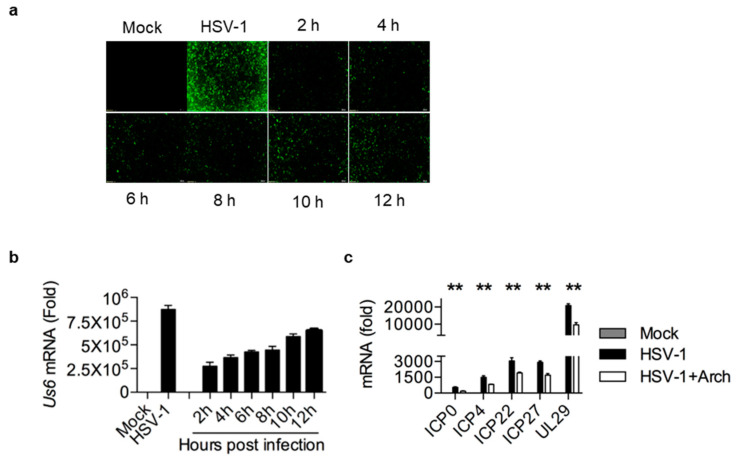
Archangiumide suppressed early viral replication. At different times after HSV-1 infection (MOI = 0.1), 0.4 mM archangiumide was added to culture systems to suppress replication. (**a**) GFP signal was captured at 24 h. Fluorescent images were in 200× magnification. (**b**) gD transcription (Us6 mRNA) was analyzed by qPCR after 24 h. Unpaired test, two-tailed. **, *p* < 0.01, n = 3. (**c**) Early-replication-related gene transcriptions (ICP0, ICP4, ICP22, ICP27, UL29 mRNA) were analyzed by qPCR before 6 h post infection. Kruskl–Wallis one-way ANOVA for each gene. n = 3. Data shown represent three independent experiments.

As archangiumide showed no effect on viral entry of cells, we next explored the timing of its antiviral effect on intracellular viruses after entry. At different time points post infection, 0.4 mM archangiumide was added to suppress HSV-1 replication. The data show that archangiumide was effective as early as 2 h post infection (Figure 4a,b), and an extremely strong antiviral effect (***, *p* < 0.001) was observed within 6 h. We next analyzed early replication genes (ICP0, ICP4, ICP22, ICP27, and UL29) and confirmed that archangiumide suppressed these genes’ transcription as predicted (Figure 4c).

### 2.5. Archangiumide Blocked VP16 Nuclear Translocation to Suppress Early Replication

**Figure 5:**
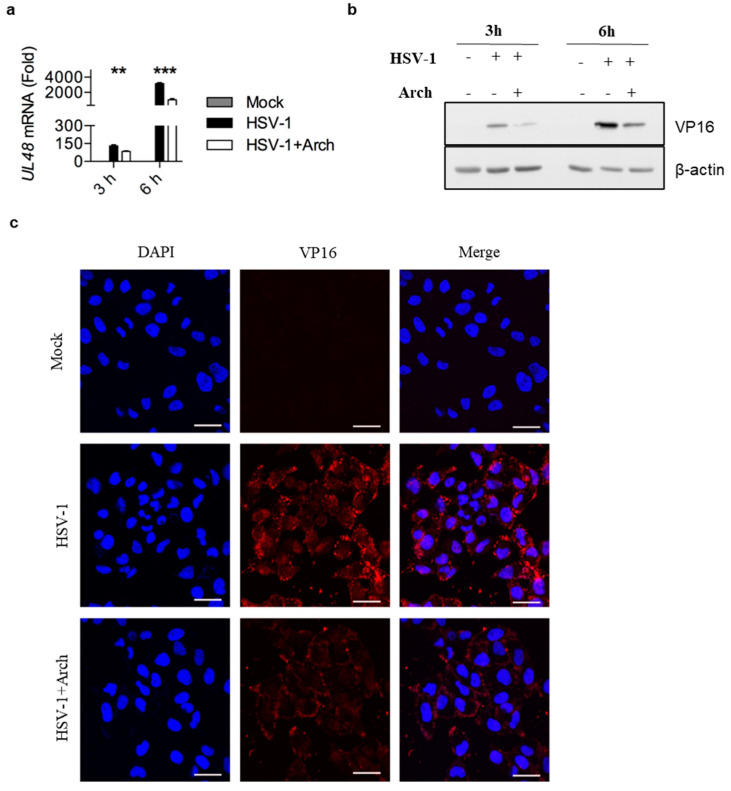
Archangiumide down-regulated VP16 expression and blocked its nuclear translocation. 0.4 mM archangiumide was added to HSV-1-infected cells (MOI = 4). (**a**) At 3 h and 6 h, VP16 (UL48 mRNA) was analyzed by qPCR. Kruskl–Wallis one-way ANOVA for each timepoint. ***, *p* < 0.001, **, *p* < 0.01, n = 3. (**b**) Western blot of VP16 protein under archangiumide. β-actin as control. (**c**) Intracellular distribution of VP16. DAPI as nuclear indicator. Bars indicate 10 μm. Data shown represent three independent experiments.

To decipher archangiumide’s early intervention in HSV-1 replication, we turned to the literature for a master regulator of early replication and focused on VP16. VP16 is well known for its transactivator function, which forms a complex with various transcriptional factors to regulate HSV-1 early replication [14,15,16,17]. Therefore, we analyzed VP16 at an early stage of infection, and found that archangiumide down-regulated VP16 mRNA and protein expression instantly (Figure 5a,b). As a transactivator, VP16’s nuclear translocation is a prerequisite for its function; therefore, we analyzed VP16’s distribution under archangiumide. The data in Figure 5c show that VP16’s nuclear translocation was blocked by archangiumide. Together, we found that archangiumide suppressed HSV-1 early replication by down-regulation of VP16’s expression and nuclear translocation.

## 3. Discussion

Here, we have reported that macrolide archangiumide suppressed HSV-1 replication in the early stages of infection. Archangiumide is a novel macrolide natural product produced by the myxobacterium archangium violaceum SDU8 [5]. Firstly, we evaluated the inhibitory activity of archangiumide on HSV-1 replication by detecting the virus titer, virus genome copy, and mRNA level. The results indicated that archangiumide blocked HSV-1 replication efficiently. The therapeutic index was calculated according to IC50 and CC50, which indicated the promising prospect of archangiumide as an anti-HSV-1 inhibitor (Figure 1). Then, we attempted to discover the mechanism by which archangiumide inhibits HSV-1 replication. Antiviral agents block virus proliferation by inactivating viral particles directly or by inhibiting the virus replication cycle. Our data demonstrate that archangiumide could not inactivate HSV-1 viral particles directly (Figure 2). That means archangiumide acted on HSV-1 replication cycle. Therefore, step by step, we scrutinized archangiumide’s intervention in HSV-1’s replication. The data indicate that archangiumide did not block HSV-1’s attachment to the cells, nor its penetration (Figure 3). We next explored the timing of its antiviral effect on intracellular viruses after entry and found that archangiumide was effective at as early as 2 h post infection, and extremely strong antiviral effect was observed within 6 h (Figure 4a,b). To decipher archangiumide’s early intervention in HSV-1 replication, we turned to the literature for a master regulator of early replication and focused on VP16. VP16 is well known for its transactivator function, which forms a complex with various transcriptional factors to regulate HSV-1 early replication [18,19,20]. Therefore, we analyzed VP16 at early stage of infection, and found that archangiumide down-regulated VP16 mRNA and protein expression instantly (Figure 5a,b). As a transactivator, VP16’s nuclear translocation is a prerequisite for its function; therefore, we analyzed VP16’s distribution under archangiumide. The data in Figure 5c show that VP16’s nuclear translocation was blocked by archangiumide. Together, we found that archangiumide suppressed HSV-1 early replication by down-regulation of VP16’s expression and nuclear translocation.

Although we have confidence in our conclusions, several limitations of this study should be acknowledged. First, as the first-ever allene bearing macrolide, whether archangiumide’s viral suppression is directly associated with the allene group is an open question, as is the question of why it did not exhibit traditional antibacterial function. As the total synthesis of this molecule has recently been mapped out [6], it is worthwhile to test these pharmaceutical questions with structural analogs without allene group. Second, although HSV-1 is the most successful virus in terms of infectivity, additional work is planned to explore more pathological viruses, such as COVID-19 and influenza. Also, deviating from HSV-1’s DNA genome nature, other RNA viruses need to be tested. Third, we presented the evidence of direct intervention of archangiumide in HSV-1 replication here. However, in vivo, more complex pharmacological and immunological effects of archangiumide are next to be explored. Last but not least, HSV-1 has a lytic-latency life cycle in vivo [11], which is the key survival technique for the pathogen. Therefore, before archangiumide’s translation from bench to bed, its impact on HSV-1 lytic-latency cycle should be monitored in animal models.

In recent years, HSV-1 infection has increased and caused a great challenge to public health. Due to drug resistance, natural products have been chosen for the development of new anti-HSV-1 agents. Many such efforts have been made [14]. For example, peniterphenyl A, from a deep-sea-derived *Penicillium* sp., showed anti-HSV-1/2 activity in vitro [21], and a hydroethanolic extract of *Tanacetum parthenium* (L.) Sch.Bip. (Asteraceae) has shown in vivo anti-HSV-1 activity and is safe for oral and topical application [22].

## 4. Materials and Methods

### 4.1. Isolation of Archangiumide

The method for the fermentation and isolation of archangiumide was described previously [11]. Archangiumide stock was prepared in 1 mM DMSO, then diluted to the desired concentration in 2% FBS/DMEM before experiments.

### 4.2. Cells and Virus

Cell passage was cultured in DMEM containing 10% FBS. Vero was cultivated to produce HSV-1-eGFP. HeLa cells lines were cultivated to perform all infection tests. HSV-1-eGFP was kindly provided by professor Xiaojia Wang (China Agricultural University, Beijing, China). For drug efficacy tests, HSV-1-eGFP stock was diluted to a certain MOI in 2% FBS/DMEM, then was allowed to infect 90% confluent HeLa cells at 37 °C. Cytopathic effects were monitored each 6 h and infected cells were typically collected for further tests at 24 h post infection.

### 4.3. TCID50 and Genome Copy

For the viral titer, samples were serial diluted from 10^−1^ to 10^−8^. The eGFP signal was captured in diluted-sample-infected cells. TCID50 was calculated using the Reed–Muench method. Eight technical repeats were performed for every diluted sample. For genome copy analysis, HSV-1 VP16 was cloned in a plasmid vector. A standard curve was generated by VP16 qPCR (ΔCT) of serial-diluted plasmid. The genome DNA of HSV-1-infected cells was extracted (Solarbio Life Sciences, Beijing, China) and then analyzed by VP16 qPCR. After fitting to the standard curve, the HSV-1 genome copy of the infected cells was calculated.

### 4.4. Cell Survival Rate

MTT-based cell viability was analyzed following the producer’s manual (Beyotime Biotechnol, Shanghai, China). Briefly, 5000 HeLa cells were seeded in 96-well plates, then archangiumide was added to the culture system for 24 h. An amount of 10 μL MTT solution (5 mg/mL) was added and cultivated at 37 °C for 4 h. An amount of 100 μL formazan solution was added and mixed gently. After 4 h, OD570 was analyzed. Four technical repeats were performed.

### 4.5. Real-Time qPCR

HSV-1 mRNA transcriptions were analyzed by RT and real-time qPCR (Mei5bio, Beijing, China). Primers used: Us6 (gD), forward, 5’-CGTCCGGAAACAACCCTACA-3’, reverse, 5’-CCCAGGTTATCCTCGCTGAC-3’. UL48 (VP16), forward, 5’-CTTCAGGTATGGCGAGTCCC-3’, reverse, 5’-GGTGTTCGTCGTCTTCGGAT-3’. ICP0, forward, 5’-GTCTGTCGCATTTGCACCTC-3’, reverse, 5’-CTTCTGTGGTGATGCCGGAG-3’. ICP4, forward, 5’-CTCCATGGTAGAGGAGGCCG-3’, reverse, 5’-CATCGTCGTCGGCTCGAAAG-3’. ICP22, forward, 5’-ATCAGCTGTTTCGGGTCCTG-3’, reverse, 5’-CCATCAGGTAACAGTCGCGT-3’. ICP27, forward, 5’-ATGTGCATCCACCACAACCT-3’, reverse, 5’-TCCTTAATGTCCGCCAGACG-3’. UL29, forward, 5’-ATGAACAGCTGCAACGGGTA-3’, reverse, 5’-GTCGTTACCGAGGGCTTCAA-3’.

### 4.6. Western Blot

Protein sample preparation and western blot were performed as instructed (Beyotime Biosciences, Beijing, China). Monoclonal antibodies against gD (sc-21719) were purchased from SANTA, VP16 (AB110226) was purchased from Abcam (Waltham, MA, USA), and β-actin (A5441) was commercially available (Sigma-Aldrich, St. Louis, MO, USA).

### 4.7. Immunofluorescence

Fixed and permeabilized cells were stained with anti-VP16 antibody (AB110226, Abcam) and then labelled with goat anti-mouse IgG (H + L)-Alexa Fluor 555 antibody (Gene Protein Link, Beijing, China). DAPI was co-stained to label nuclei. Fluorescent images were captured with an A1-SIM Laser Scanning Confocal Microscope (Nikon, Minato City, Japan).

## Data Availability

Data are contained within the article.

## References

[1] Dinos G.P. (2017). The macrolide antibiotic renaissance. Br. J. Pharmacol..

[2] Fernandes P., Martens E., Pereira D. (2017). Nature nurtures the design of new semi-synthetic macrolide antibiotics. J. Antibiot..

[3] Myers A.G., Clark R.B. (2021). Discovery of Macrolide Antibiotics Effective against Multi-Drug Resistant Gram-Negative Pathogens. Acc. Chem. Res..

[4] Kim K., Jung S., Kim M., Park S., Yang H.J., Lee E. (2022). Global Trends in the Proportion of Macrolide-Resistant Mycoplasma pneumoniae Infections: A Systematic Review and Meta-analysis. JAMA Netw. Open.

[5] Hu J.-Q., Wang J.-J., Li Y.-L., Zhuo L., Zhang A., Sui H.-Y., Li X.-J., Shen T., Yin Y., Wu Z.-H. (2021). Combining NMR-Based Metabolic Profiling and Genome Mining for the Accelerated Discovery of Archangiumide, an Allenic Macrolide from the Myxobacterium Archangium violaceum SDU8. Org. Lett..

[6] Sutro J.L., Furstner A. (2024). Total Synthesis of the Allenic Macrolide (+)-Archangiumide. J. Am. Chem. Soc..

[7] Batiha GE S., Zayed M.A., Awad A.A., Shaheen H.M., Mustapha S., Herrera-Calderon O., Pagnossa J.P., Algammal A.M., Zahoor M., Adhikari A. (2021). Management of SARS-CoV-2 Infection: Key Focus in Macrolides Efficacy for COVID-19. Front. Med..

[8] Pani A., Lauriola M., Romandini A., Scaglione F. (2020). Macrolides and viral infections: Focus on azithromycin in COVID-19 pathology. Int. J. Antimicrob. Agents.

[9] Khoshnood S., Shirani M., Dalir A., Moradi M., Haddadi M.H., Sadeghifard N., Birjandi F.S., Yashmi I., Heidary M. (2022). Antiviral effects of azithromycin: A narrative review. Biomed. Pharmacother..

[10] Shushni M.A.M., Singh R., Mentel R., Lindequist U. (2011). Balticolid: A new 12-membered macrolide with antiviral activity from an ascomycetous fungus of marine origin. Mar. Drugs.

[11] Amin I., Vajeeha A., Younas S., Afzal S., Shahid M., Nawaz R., Khan M.U., Idrees M. (2019). HSV-1 Infection: Role of Viral Proteins and Cellular Receptors. Crit. Rev. Eukaryot. Gene Expr..

[12] Zhu S., Viejo-Borbolla A. (2021). Pathogenesis and virulence of herpes simplex virus. Virulence.

[13] Ahmad I., Wilson D.W. (2020). HSV-1 Cytoplasmic Envelopment and Egress. Int. J. Mol. Sci..

[14] Treml J., Gazdova M., Smejkal K., Sudomova M., Kubatka P., Hassan S.T.S. (2020). Natural Products-Derived Chemicals: Breaking Barriers to Novel Anti-HSV Drug Development. Viruses.

[15] Yu W., Geng S., Suo Y., Wei X., Cai Q., Wu B., Zhou X., Shi Y., Wang B. (2018). Critical Role of Regulatory T Cells in the Latency and Stress-Induced Reactivation of HSV-1. Cell Rep..

[16] Pietila M.K., Bachmann J.J., Ravantti J., Pelkmans L., Fraefel C. (2023). Cellular state landscape and herpes simplex virus type 1 infection progression are connected. Nat. Commun..

[17] Reske A., Pollara G., Krummenacher C., Chain B.M., Katz D.R. (2007). Understanding HSV-1 entry glycoproteins. Rev. Med. Virol..

[18] Wysocka J., Herr W. (2003). The herpes simplex virus VP16-induced complex: The makings of a regulatory switch. Trends Biochem. Sci..

[19] Herrera F.J., Triezenberg S.J. (2004). VP16-dependent association of chromatin-modifying coactivators and underrepresentation of histones at immediate-early gene promoters during herpes simplex virus infection. J. Virol..

[20] Triezenberg S.J., Kingsbury R.C., McKnight S.L. (1988). Functional dissection of VP16, the trans-activator of herpes simplex virus immediate early gene expression. Genes Dev..

[21] Chen W., Zhang J., Qi X., Zhao K., Pang X., Lin X., Liao S., Yang B., Zhou X., Liu S. (2021). p-Terphenyls as Anti-HSV-1/2 Agents from a Deep-Sea-Derived Penicillium sp.. J. Nat. Prod..

[22] Benassi-Zanqueta E., Marques C.F., Valone L.M., Pellegrini B.L., Bauermeister A., Ferreira I.C.P., Lopes N.P., Nakamura C.V., Filho B.P.D., Natali M.R.M. (2019). Evaluation of anti-HSV-1 activity and toxicity of hydroethanolic extract of *Tanacetum parthenium* (L.) Sch.Bip. (Asteraceae). Phytomedicine.

